# Mild Hyperthermia-Induced Myogenic Differentiation in Skeletal Muscle Cells: Implications for Local Hyperthermic Therapy for Skeletal Muscle Injury

**DOI:** 10.1155/2018/2393570

**Published:** 2018-06-27

**Authors:** Hojun Lee, Seung-Jun Choi

**Affiliations:** ^1^Mechanical & Molecular Myology Lab, Department of Rehabilitation Medicine, College of Medicine, Seoul National University Bundang Hospital, Seongnam, Republic of Korea; ^2^Division of Sports and Health Science, Kyungsung University, Busan, Republic of Korea

## Abstract

The percutaneous application of controlled temperature on damaged muscle is regarded as a prevalent remedy. However, specific mechanisms are not completely understood. Therefore, cellular behaviors of myoblasts were investigated under a physiological hyperthermic temperature. The myoblasts were cultured under no treatment (NT, 37°C, 24 h/day), intermittent heat treatment (IHT, 39°C, 2 h/day), and continuous heat treatment (CHT, 39°C, 24 h/day) during proliferation, migration, or myogenic differentiation. Although the effects of mild heat on migration were not observed, the proliferation was promoted by both IHT and CHT. The myogenic differentiation was also enhanced in a treatment time-dependent manner, as evidenced by an increase in myotube size and fusion index. The gene expressions of mitochondrial biogenesis (Pgc-1*α*, Nrf1, and Tfam), a subset of mitochondrial dynamics (Mfn1 and Drp1), and a myogenic regulatory factor (myogenin) were increased in a heat treatment time-dependent manner. Interestingly, the mild heat-induced myogenic differentiation and myogenin expression were retarded significantly in PGC-1*α*-targeted siRNA-transfected cells, suggesting that mild hyperthermia promotes myogenic differentiation via the modulation of PGC-1*α*. This study provides cellular evidence supporting that local hyperthermic treatment at 39°C is regarded as an effective therapeutic strategy to promote satellite cell activities in regenerating myofibers.

## 1. Introduction

Recovery from skeletal muscle injury is orchestrated by a series of events consisting of degeneration, inflammation, and regeneration [1]. In the early phase of muscle recovery, significant destruction of myofibers called necrosis occurs, which is immediately followed by the removal of damaged cellular debris [2]. It is reported that, based on the level of injury state, the degeneration and inflammatory phase last up to 1 to 7 days [3, 4]. During the inflammatory stage, macrophages matured from monocytes not only remove damaged cells by phagocytosis but produce chemotactic signaling molecules leading to the activation of satellite cells for the proper regeneration of injured myofibers [3].

In the field of rehabilitation and sports medicine, there has been a great deal of effort in promoting skeletal muscle recovery. The most prevalent noninvasive strategy is the local application of controlled temperature on the damaged skeletal muscle. In the past, the local hypothemic therapy was regarded to be an effective strategy in the management of injury-induced pain, swelling, and inflammatory reactions. [5–8]. However, a study suggested that skeletal muscle regeneration is attenuated by hypothemic therapy due to the excessive suppression of inflammatory reactions, suggesting that a proper inflammatory process in the initial phase of recovery is critical for the facilitated regeneration of myofibers [9]. In line with this, heat treatment has been demonstrated to increase macrophage activation, leading to an increase in the number of regenerating muscle fibers in the damaged extensor digitorum longus of experimental rats [4]. In addition, several lines of studies proved that the application of mild heat treatment at 39°C is conducive to myogenesis while temperature at 41°C is detrimental to satellite cell activities [10, 11], providing a piece of evidence that the intrinsic biological capacity of satellite cell is tightly modulated in a temperature-sensitive manner.

Satellite cells are multipotent myogenic stem cells and exist between the sarcolemma and the basement membrane of myofibers [12]. Satellite cell-mediated muscle regeneration involves the orchestration of a series of cellular processes. Specifically, once activated, quiescent satellite cells enter proliferation phase and migrate to the site of injury, followed by myogenic differentiation to form new fibers or to incorporate into existing fibers [13, 14], all which steps are critical for the proper remodeling of the skeletal muscle. However, specific mechanisms of heat-mediated myogenic differentiation are not fully understood.

To elucidate cellular behaviors of myoblasts, cells were cultured under three different conditions in the study. The value of chronic heat treatment (CHT) was set at 39°C since this temperature can be easily reached in deep skeletal muscles under physiological systems [15]. In comparison, the myoblasts (IHT) were subjected to a cycle of 2 hour 39°C and 22 hour 37°C until being assayed. The heat-treated cells were compared with the cells cultured at 37°C.

## 2. Materials and Methods

### 2.1. Cell Culture

C2C12 myoblasts were seeded onto collagen-coated 6-well plates and were maintained in Dulbecco's Modified Eagle's Medium (Welgen, Kyungsan, Korea) containing 10% fetal bovine serum (FBS), 100 units/mL penicillin, and 100 mg/mL streptomycin (Welgene, Kyungsan, Korea) in a humidified atmosphere of 5% CO_2_ at 37°C or 39°C. When the myoblasts were confluent (95%), growth medium (10% FBS) was changed to differentiation medium (DM) supplemented with 2% horse serum, 100 units/mL penicillin, and 100 mg/mL streptomycin (Welgene, Kyungsan, Korea). Cells were differentiated for 3 to 5 days at 37°C or 39°C. For chronic heat treatment, proliferating or differentiating myoblasts were incubated at 39°C continuously for the indicated time. For intermittent heat treatment (IHT), proliferating or differentiating myoblasts were incubated for 2 hours at 39°C, followed by 22 hours at 37°C a day. This cycle was repeated until the end of each experimental protocol. The specific incubation time for each experiment is described in each figure legend. Cell culture was performed in a minimum of four independent times. siRNA transfection was performed as previously described [16]. Briefly, Pgc-1*α* siRNA or scrambled siRNA (Santa Cruz Biotechnology, CA, USA) was prediluted in OPTI-MEM medium (Gibco, MD, USA) containing Lipofectamine 2000 (Gibco, MD, USA). Lipofectamine-siRNA complexes were added into each well immediately after cells were plated in DMEM + 5% FBS without 100 units/mL penicillin, and 100 mg/mL streptomycin (final concentration of each siRNA was 10 nM). After 6 h of incubation, the media was exchanged with DMEM supplemented with 10% FBS for 18 h.

### 2.2. Cell Counting Assay

Cell counting assay was conducted using the trypan blue exclusion assay with minor modification [17]. C2C12 myoblasts were plated onto 60 mm culture dishes at 30% confluence and grown in growth medium at the indicated temperatures. Medium was changed every 24 h for 48 hours. The cells were trypsinized and stained with trypan blue. The nonstained viable cells were counted using a hemacytometer. The number of cells was expressed as myoblast number/mL.

### 2.3. Myoblast Migration Assay

An acellular area was generated in 95% confluent monolayers of myoblasts by scraping with a 200 *μ*L pipette tip. Myoblast migration test was performed in a culture medium (DMEM) supplemented with 0.1% FBS and 100 units/mL penicillin and 100 mg/mL streptomycin for 24 hours in a humidified atmosphere of 5% CO_2_ at the indicated temperature conditions. The myoblasts that migrated into the acellular area were measured. The images were acquired using a phase contrast microscope (Carl Zeiss, Jena, Germany) 24 hours after the generation of the cellular area.

### 2.4. Immunocytochemistry for Myosin Heavy Chain and Myonuclei

Differentiated myotubes were fixed and permeabilized by incubating in ice-cold methanol (Sigma-Aldrich, Saint Louis, USA) for 10 minutes. After rehydration in Dulbecco's phosphate-buffered saline (DPBS) three times, the myotubes were incubated in blocking solution including 2% bovine serum albumin (BSA) for 30 minutes at room temperature, followed by incubation in 2% BSA solution containing anti-sarcomeric myosin antibody MF-20 conjugated with Alexa Fluor 488 (eBioscience, San Diego, USA). After myonuclei were stained with 4′,6-diamidino-2-phenylindole (DAPI) (Molecular Probes, Eugene, OR, USA), the micrographs were acquired under a fluorescence microscope (Carl Zeiss, Jena, Germany).

### 2.5. Fusion Index

Fusion index is a myological indicator showing the degree of myogenic differentiation to myotubes. The index is calculated as the number of nuclei in myotubes divided by the total number of nuclei observed in the field, which is expressed as %. The cells with a minimum number of three myonuclei were counted as differentiated myotubes.

### 2.6. qRT-PCR

Differentiated myotubes were washed with cold DPBS and lysed with TRIzol reagent. Chloroform was added for the separation of RNA from DNA and protein fractions. The RNA fraction was precipitated by the addition of isopropanol, followed by centrifugation at 12,000*g* for 8 minutes at 4°C. The RNA pellet was washed with 75% ethanol and centrifuged at 12,000*g* for 5 minutes at 4°C. After removal of the ethanol, the RNA pellets were air-dried before resuspending with RNAse free water. The RNA concentration was quantified using a NanoDrop spectrophotometer (NanoDrop Technologies, DE, USA). Reverse transcription was performed with a cDNA synthesis kit according to manufacturer's instruction (Bioneer, Daejeon, Korea). Following complementary DNA synthesis, qRT-PCR was performed in a Real-Time PCR system (Applied Biosystems, CA, US) using a SYBR Green Master Mix (Bioline, Hanam, Korea), and the primer pair sets were described in Table 1. Cycle threshold (Ct) values were normalized to the housekeeping gene (HPRT1-F: 5′- GACTTGCTCGAGATGTCATG -3′, HPRT1-R: 5′- TACAGTCATAGGAATGGACC -3′).

### 2.7. Statistical Analysis

The results were presented as mean ± SEM for a minimum of four independent experiments. One-way ANOVA was followed by Tukey's post hoc test to present statistical difference among groups. The statistical significance was set at *p* < 0.05.

## 3. Results

### 3.1. Effects of IHT and CHT on Migration and Proliferation of Myoblasts

**Figure 1:**
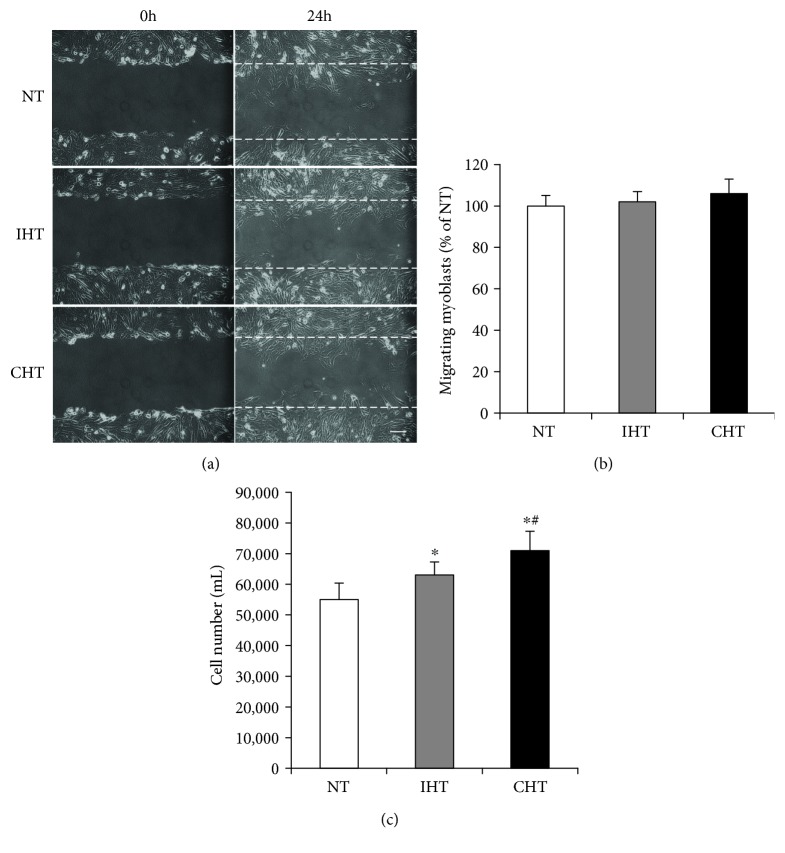
Mild heat treatment promotes myoblast proliferation but not migration. (a and b) Migrating myoblasts were observed under phase contrast microscope (magnification = 20x). Scale bar represents 50 *μ*m. After scratch onto 60 mm culture plates with a 200 *μ*m pipette tip at 95% confluency, migrating myoblasts were measured 24 hours after scratch and were expressed as % of NT. (c) The trypsin-treated myoblasts suspended in medium were counted with hemocytometer 48 hours after incubation in DMEM supplemented with 10% FBS under specified temperature condition. The data were expressed as cells/mL. Each experiment was performed with at least four separate cell cultures. The bar graph represented means ± SEM. The data were analyzed using one-way ANOVA followed by Tukey's post hoc test. ^∗^*p* < 0.05 versus NT (no treatment); ^#^*p* < 0.05 versus IHT (intermittent heat treatment).

The migrating myoblasts (%) were not changed statistically in the IHT and CHT groups compared to NT (Figures 1(a) and 1(b)). The myoblast proliferation was increased by the IHT and CHT in a treatment time-dependent manner (*p* < 0.05, Figure 1(c))

### 3.2. Effects of IHT and CHT on Myogenic Differentiation

**Figure 2:**
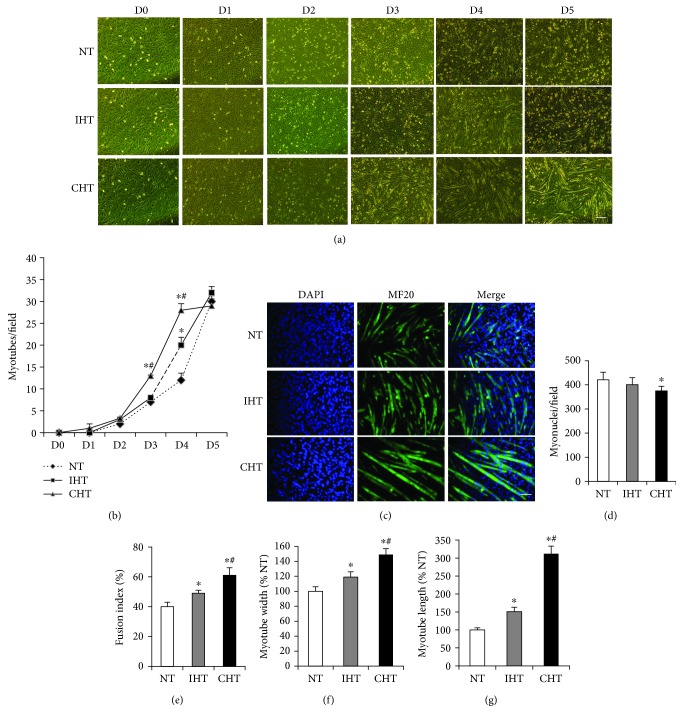
Mild heat treatment promotes myogenic differentiation in a treatment time-dependent manner. (a) Myoblasts were differentiated in DMEM supplemented with 2% horse serum under specified temperature condition for 5 days. Differentiating myoblasts were observed and counted every 24 hours under phase contrast microscope (magnification = 10x). Scale bar represents 100 *μ*m. (b) Myotubes were counted and expressed as number per field. (c) Differentiated myotubes were stained with DAPI and MF20 antibody and were visualized by fluorescence microscope (magnification = 20x). Scale bar represents 50 *μ*m. (d) Myonuclei, (e) fusion index, (f) myotube width, (g) and myotube length were quantified. Each experiment was performed with at least four separate cell cultures. Bar graph represented means ± SEM. The data were analyzed using one-way ANOVA followed by Tukey's post hoc test. ^∗^*p* < 0.05 versus NT (no treatment); ^#^*p* < 0.05 versus IHT (intermittent heat treatment).

On day 3, the number of myotubes formed during differentiation was higher in the CHT group compared to the IHT and NT groups (*p* < 0.05, Figures 2(a) and 2(b)). On day 4, the number of myotubes was higher in the IHT group compared to NT group while the number was the highest in the CHT group (Figures 2(a) and 2(b)). To explore myotube morphology in depth, the myosin heavy chain and myonuclei in myotubes differentiated for 4 days were stained with MF20 and DAPI (4′,6-diamidino-2-phenylindole) (Figure 2(b)). The total myonuclei number was lower in the CHT group compared to the IHT and NT groups (Figure 2(d)) while the myotube fusion index (calculated as the number of nuclei in myotubes divided by the total number of nuclei counted) was greatest in the CHT group (*p* < 0.05, Figure 2(e)), indicating that more myoblasts were involved in myogenic differentiation. The width and length of myotubes were the greatest in the CHT, followed by the IHT and NT (*p* < 0.05, Figures 2(f) and 2(g)).

### 3.3. Effects of IHT and CHT on the Gene Expressions of Mitochondrial Remodeling

**Figure 3:**
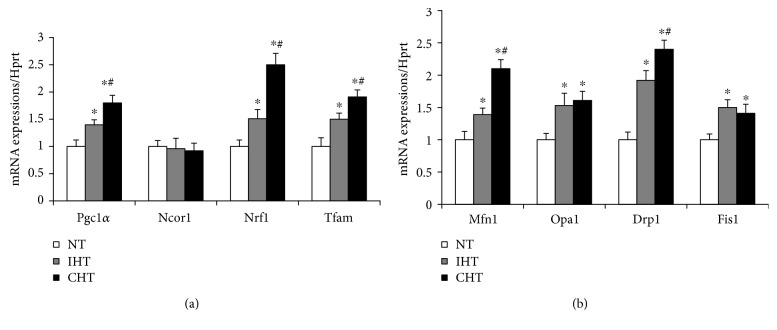
Mild heat treatment promotes mitochondrial remodeling in a treatment time-dependent manner. Myoblasts were differentiated in DMEM supplemented with 2% horse serum under specified temperature condition. (a) The gene expressions of positive regulators (Pgc-1*α*, Nrf1, and Tfam) and a negative regulator (Ncor1) of mitochondrial biogenesis were quantified. (b) The gene expressions of mitochondrial fusion (Mfn1 and Opa1) and fission (Drp1 and Fis1) were quantified. Each experiment was performed with at least four separate cell cultures. Bar graph represented means ± SEM. The data were analyzed using one-way ANOVA followed by Tukey's post hoc test. ^∗^*p* < 0.05 versus NT (no treatment); ^#^*p* < 0.05 versus IHT (intermittent heat treatment).

The multiple gene expressions of mitochondrial biogenesis (Pgc1-*α*, Nrf1, and Tfam) were increased in a treatment time-dependent manner (*p* < 0.05, Figure 3(a)). The expression of Ncor1, a negative regulator of mitochondrial biogenesis, was not changed by both treatments (Figure 3(a)). The gene expressions of all the mitochondrial dynamics markers (Mfn1, Opa1, Drp1, and Fis1) were increased by both treatment methods. Specifically, the expressions of Mfn1 and Drp1 were the greatest in the CHT group, followed by the IHT and NT group (*p* < 0.05, Figure 3(b)).

### 3.4. Effects of IHT and CHT on the Gene Expressions of Myosin Heavy Chain Isoforms and Myogenin

**Figure 4:**
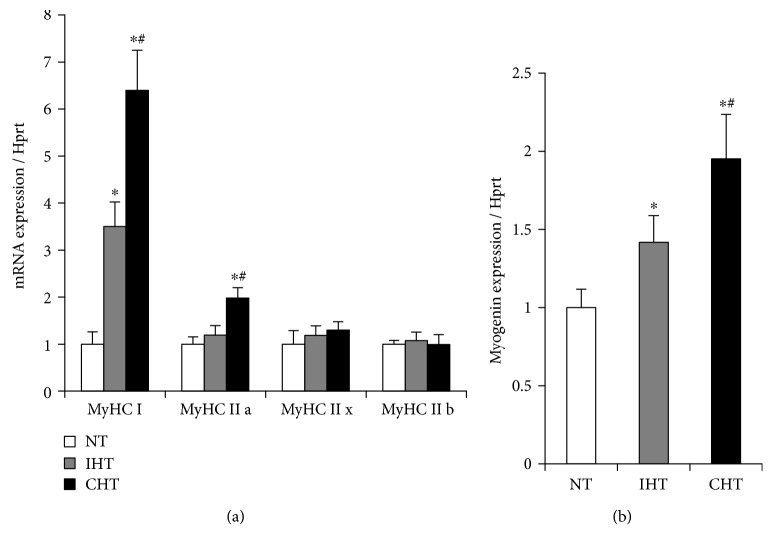
Mild heat treatment promotes the expressions of MyHCI and myogenin in a treatment time-dependent manner. (a) The gene expressions of myosin heavy chain isoforms (MyHCI, IIa, IIx, and IIb) and (b) myogenic factor (myogenin) were quantified in myotubes differentiated under specified temperature condition. Each experiment was performed with at least four separate cell cultures. Bar graph represented means ± SEM. The data were analyzed using one-way ANOVA followed by Tukey's post hoc test. ^∗^*p* < 0.05 versus NT (no treatment); ^#^*p* < 0.05 versus IHT (intermittent heat treatment).

The gene expressions of myosin heavy chain isoforms and myogenic markers were measured. The expression of MyHCI was increased in both treatment groups in a treatment time-dependent manner. The expression of MyHCIIa was increased only in the CHT group compared to NT group (*p* < 0.05, Figure 4(a)). The expression of MyHCIIx and IIb were not altered by both treatments. The expression of myogenin was increased in a treatment time-dependent manner (*p* < 0.05, Figure 4(b)).

### 3.5. PGC-1*α*-Dependent Mild Heat-Induced Myogenic Differentiation

**Figure 5:**
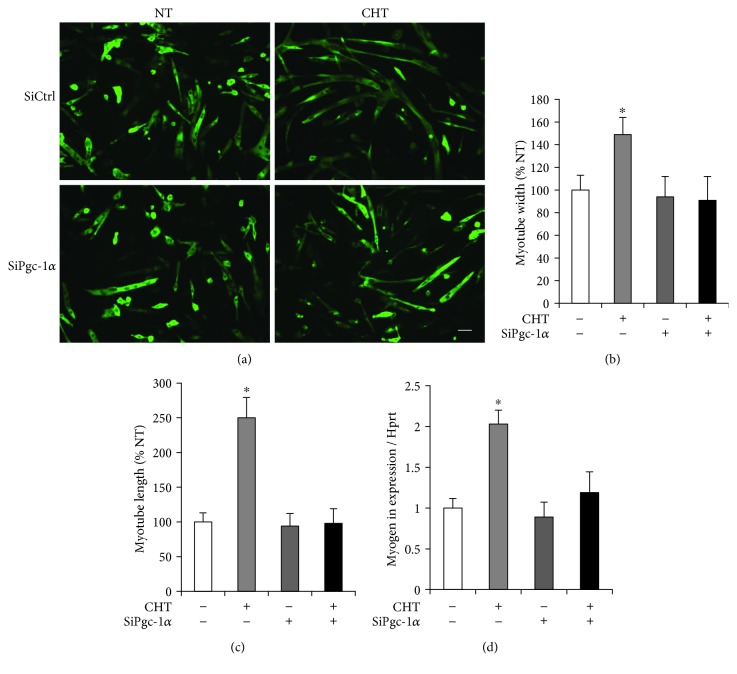
Mild heat treatment promotes myogenic differentiation in a PGC-1*α* dependent manner. (a) Myoblasts were transfected with control siRNA or Pgc-1*α* siRNA and were incubated to differentiate for 72 hours under specified temperature condition in DMEM supplemented with 2% horse serum, 100 units/mL penicillin, and 100 mg/mL streptomycin. Differentiated myotubes were stained with DAPI and were visualized by fluorescence microscope (magnification = 20x). Scale bar represents 50 *μ*m. (b) Myotube width, (c) myotube length, and (d) the levels of myogenic expression were quantified. Each experiment was performed with at least four separate cell cultures. Bar graph represented means ± SEM. The data were analyzed using one-way ANOVA followed by Tukey's post hoc test. ^∗^*p* < 0.05 versus untreated cells.

The mild heat-induced width and length of myotubes and myogenin expression were decreased to the level of nonheat-treated cells in the PGC-1*α*-targeted siRNA-transfected cells (*p* < 0.05, Figure 5).

## 4. Discussion

The application of controlled temperature on injury site has been regarded as a prevalent noninvasive method for myofiber regeneration [8, 18]. However, cellular mechanisms are not completely understood. Here, we demonstrated that mild hyperthermic treatment promotes myoblast proliferation and myogenic differentiation in a treatment time-dependent manner, but not migration. Our results also suggested that mild heat-induced myogenic differentiation is controlled in PGC-1*α*-dependent manner.

Satellite cells exist in a sublaminar niche in a mitotically quiescent state under basal conditions [19]. Once activated by injury-induced extracellular stimuli such as hepatocyte growth factor and nitric oxide, quiescent satellite cells enter the phase of self-renewing process to transform to proliferating myogenic cells called myoblasts [20]. In the current study, the mild hyperthermic treatments stimulated the rate of myoblasts proliferation. This result is in accordance with a previous study reporting that nuclear DNA contents, a surrogate marker of proliferation, were increased in a temperature-sensitive manner [21], suggesting a possibility that mild heat treatment at 39°C promotes an initial step of satellite self-renewing process. This idea is further supported by a rodent study demonstrating that the percutaneous application of a 20 minute heat treatment, immediately after injury, to the extensor digitorum longus (EDL) increased the number of regenerating fibers [4].

Satellite cells move along myofibers in an external chemotactic molecule-dependent manner [22]. Since satellite cells are sparsely scattered along the myofiber, an efficient motility of myoblasts is warranted for rapid myofiber recovery [23]. In the present study, the hyperthermic treatment did not change the rate of myoblast migration (Figures 1(a) and 1(b)). This suggests that hyperthermia alone is not sufficient to accelerate the motility of satellite cells. Currently, the effect of mild heat treatment on myoblast migration has not been reported elsewhere. As it has been known that the rate of myoblast migration is significantly affected by external molecular signals such as HGF, FGF, and SDF-1 [22], future studies are warranted to test if heat treatment would have synergistic effects on satellite cell migration under the treatment of known external chemoattractants.

Myoblast fusion is initiated by myogenin-induced myogenic differentiation to incorporate into myofibers, which is considered a critical and irreversible step in satellite cell-mediated muscle regeneration [19]. In the present study, a greater number of myotubes was observed in both CHT and IHT cells compared to NT at the fourth day of differentiation. The increased myogenic differentiation is further supported by the enhanced fusion index and myotube sizes in a heat treatment time-dependent fashion (Figures 2(a)–2(g)). In previous *in vivo* studies using rodents, the effects of hyperthermia have been demonstrated by immersing animals with injured legs into warm water for only 20 to 30 minutes a day to avoid the excessive increase of the body temperature [4, 24, 25]. Therefore, it still remains elusive if a longer treatment would further enhance the rate of skeletal muscle regeneration. Regarding this notion, our results imply a piece of cellular possibility that, at a phase of myogenic differentiation, the duration of local hyperthermic therapy to injured skeletal muscle could be extended to facilitate the rate of satellite cell differentiation in regenerating myofibers.

Mitochondria are dynamic subcellular organelles regulating their size and mass as necessary, and their biogenesis and remodeling through fusion and fission are essential to maintain metabolic capacity in myofibers [26–28]. In this study, the hyperthermic treatments increased the expressions of mitochondrial biogenic (Pgc-1*α*, Nrf1, and Tfam) and remodeling (Mfn1, Opa1, Drp1, and Fis1) markers (Figures 3(a) and 3(b)). In line with this, multiple studies proved that mitochondrial biogenesis and remodeling of myoblasts are essential steps in myogenic differentiation [29, 30]. This suggests that metabolic shift to mitochondrial oxidative phosphorylation take a central role in mild heat-mediated myogenic differentiation. This notion is further supported by the enhanced expressions of mitochondria-rich oxidative fibers (MyHCI and MyHCIIa) and myogenic regulatory factor (myogenin) in the heat-treated cells (Figure 4). Of the myogenic regulatory factors, myogenin has been known not only to initiate the processes of myogenesis but also to act as a significant determinant to myofiber characteristics. Specifically, it has been documented that myogenin is predominately expressed in slow-twitch muscles [31, 32], indicating a distinctive role of myogenin in transforming muscle fiber to more oxidative fiber type. As the myogenin was expressed in a treatment time-dependent manner, it appears that myogenin plays a significant role in increasing the expressions of more oxidative fibers (MyHCI and MyHCIIa) during heat-mediated myogenesis.

It has been widely accepted that PGC-1*α*, a master regulator of mitochondrial biogenesis, plays a central role in a regulatory network controlling numerous mitochondria-related transcription factors during myogenic differentiation [33, 34]. As it has been proven that depletion of PGC-1*α* attenuates myogenin expression [35], myoblasts were transfected with PGC-1*α* siRNA and differentiated under hyperthermic condition to investigate a possible mechanism of heat-mediated myogenic differentiation. Interestingly, the mild heat-induced differentiation potentials were significantly attenuated in the PGC-1*α*-targeted siRNA-transfected cells (Figures 5(a)–5(c)). This is consistent with the reduced expressions of myogenin under heat treatment in the transfected cells (Figure 5(d)), indicating that mild heat-induced myogenic differentiation is controlled via the modulation of PGC-1*α* and myogenin axis.

**Figure 6:**
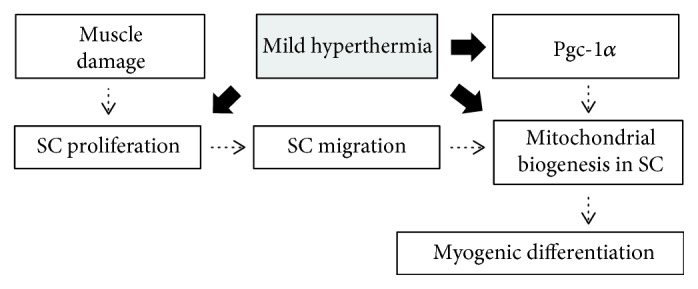
A proposed mechanism for satellite cell-mediated myogenic regeneration under mild hyperthermic condition. The dotted arrows indicate cellular processes of satellite cell-mediated myofiber regeneration. The filled arrows indicate the effect of mild hyperthermic treatment. SC: satellite cells.

In summary, this study demonstrated that mild heat treatment promotes the rate of proliferation and myogenic differentiation in a treatment time-dependent manner, which is regarded to provide an important piece of cellular evidence that clinical local hyperthermic therapy could be considered as an effective strategy to enhance the rate of regenerating myofibers (Figure 6). However, on the interpretation of our study, it should be noted that the temperature was set at 39°C as a mild hyperthermic condition. Given that the temperature in skeletal muscles could be elevated up to 44°C during hyperthermia and myogenic differentiation potential is significantly dysregulated at 41°C [36], a careful consideration should be taken into account to develop an effective hyperthermic therapy.

## Figures and Tables

**Table 1:** Primer sets for qRT-PCR.

Gene		Primer sequences
Pgc-1*α*	Forward	TGATGTGAATGACTTGGATACAGACA
	Reverse	GCTCATTGTTGTACTGGTTGGATATG
Ncor1	Forward	GACCCGAGGGAAGACTACCATT
	Reverse	ATCCTTGTCCGAGGCAATTTG
Nrf1	Forward	GAACGCCACCGATTTCACTGTC
	Reverse	CCCTACCACCCACGAATCTGG
Tfam	Forward	CTGATGGGTATGGAGAAGGAGG
	Reverse	CCAACTTCAGCCATCTGCTCTTC
Mfn1	Forward	TTGATCGAATAGCATCCGAGGA
	Reverse	CACAGCATTGCATTGATGACAG
Opa1	Forward	GGTCACCACGAGAAATCTCAG
	Reverse	TCTTCCATTCCGTCTCTAGGTT
Drp1	Forward	CGGGACAAGTTAATTCAGGACA
	Reverse	GTTCTCGGGCAGACAGTTTTC
Fis1	Forward	GCTCTAAAGTATGTGCGAGGG
	Reverse	TGCCTACCAGTCCATCTTTCTT
Myogenin	Forward	CCTTGCTCAGCTCCCTCA
	Forward	TGGGAGTTGCATTCACTGG
HPRT	Forward	GACTTGCTCGAGATGTCATG
	Reverse	TACAGTCATAGGAATGGACC

## Data Availability

The data used to support the findings of this study are available from the corresponding author upon request.

## References

[1] Quintero A. J., Wright V. J., Fu F. H., Huard J. (2009). Stem cells for the treatment of skeletal muscle injury. *Clinics in Sports Medicine*.

[2] Järvinen T. A. H., Järvinen T. L. N., Kääriäinen M., Kalimo H., Järvinen M. (2005). Muscle injuries: biology and treatment. *The American Journal of Sports Medicine*.

[3] Tidball J. G. (2005). Inflammatory processes in muscle injury and repair. *American Journal of Physiology. Regulatory, Integrative and Comparative Physiology*.

[4] Takeuchi K., Hatade T., Wakamiya S., Fujita N., Arakawa T., Miki A. (2014). Heat stress promotes skeletal muscle regeneration after crush injury in rats. *Acta Histochemica*.

[5] Michlovitz S., Smith W., Watkins M. (1988). Ice and high voltage pulsed stimulation in treatment of acute lateral ankle sprains. *The Journal of Orthopaedic and Sports Physical Therapy*.

[6] Smith T. L., Curl W. W., Smith B. P. (1993). New skeletal muscle model for the longitudinal study of alterations in microcirculation following contusion and cryotherapy. *Microsurgery*.

[7] Deal D. N., Tipton J., Rosencrance E., Curl W. W., Smith T. L. (2002). Ice reduces edema. a study of microvascular permeability in rats. *The Journal of Bone and Joint Surgery-American volume*.

[8] Bleakley C., McDonough S., MacAuley D. (2004). The use of ice in the treatment of acute soft-tissue injury: a systematic review of randomized controlled trials. *The American Journal of Sports Medicine*.

[9] Takagi R., Fujita N., Arakawa T., Kawada S., Ishii N., Miki A. (2011). Influence of icing on muscle regeneration after crush injury to skeletal muscles in rats. *Journal of Applied Physiology*.

[10] Guo Q., Miller D., An H. (2016). Controlled heat stress promotes myofibrillogenesis during myogenesis. *PLoS One*.

[11] Ikeda K., Ito A., Sato M., Kanno S., Kawabe Y., Kamihira M. (2017). Effects of heat stimulation and l-ascorbic acid 2-phosphate supplementation on myogenic differentiation of artificial skeletal muscle tissue constructs. *Journal of Tissue Engineering and Regenerative Medicine*.

[12] Zammit P. S., Partridge T. A., Yablonka-Reuveni Z. (2006). The skeletal muscle satellite cell: the stem cell that came in from the cold. *The Journal of Histochemistry and Cytochemistry*.

[13] Relaix F., Zammit P. S. (2012). Satellite cells are essential for skeletal muscle regeneration: the cell on the edge returns centre stage. *Development*.

[14] Kawano F., Takeno Y., Nakai N. (2008). Essential role of satellite cells in the growth of rat soleus muscle fibers. *American Journal of Physiology. Cell Physiology*.

[15] Wirth V. J., van Lunen B., Mistry D., Saliba E., McCue F. (1998). Temperature changes in deep muscles of humans during upper and lower extremity exercise. *Journal of Athletic Training*.

[16] Srikuea R., Zhang X., Park-Sarge O. K., Esser K. A. (2012). VDR and CYP27B1 are expressed in C2C12 cells and regenerating skeletal muscle: potential role in suppression of myoblast proliferation. *American Journal of Physiology. Cell Physiology*.

[17] Duttaroy A., Qian J. F., Smith J. S., Wang E. (1997). Up-regulated P21^CIP1^ expression is part of the regulation quantitatively controlling serum deprivation-induced apoptosis. *Journal of Cellular Biochemistry*.

[18] Taylor B. F., Waring C. A., Brashear T. A. (1995). The effects of therapeutic application of heat or cold followed by static stretch on hamstring muscle length. *The Journal of Orthopaedic and Sports Physical Therapy*.

[19] Le Grand F., Rudnicki M. A. (2007). Skeletal muscle satellite cells and adult myogenesis. *Current Opinion in Cell Biology*.

[20] Shi X., Garry D. J. (2006). Muscle stem cells in development, regeneration, and disease. *Genes & Development*.

[21] Harding R. L., Halevy O., Yahav S., Velleman S. G. (2016). The effect of temperature on proliferation and differentiation of chicken skeletal muscle satellite cells isolated from different muscle types. *Physiological Reports*.

[22] Siegel A. L., Atchison K., Fisher K. E., Davis G. E., Cornelison D. D. W. (2009). 3D timelapse analysis of muscle satellite cell motility. *Stem Cells*.

[23] Bischoff R. (1997). Chemotaxis of skeletal muscle satellite cells. *Developmental Dynamics*.

[24] Shibaguchi T., Sugiura T., Fujitsu T. (2016). Effects of icing or heat stress on the induction of fibrosis and/or regeneration of injured rat soleus muscle. *The Journal of Physiological Sciences*.

[25] Yoshihara T., Naito H., Kakigi R. (2013). Heat stress activates the Akt/mTOR signalling pathway in rat skeletal muscle. *Acta Physiologica*.

[26] Garnier A., Fortin D., Zoll J. (2005). Coordinated changes in mitochondrial function and biogenesis in healthy and diseased human skeletal muscle. *The FASEB Journal*.

[27] Ding H., Jiang N., Liu H. (2010). Response of mitochondrial fusion and fission protein gene expression to exercise in rat skeletal muscle. *Biochimica et Biophysica Acta (BBA) - General Subjects*.

[28] Yan Z., Lira V. A., Greene N. P. (2012). Exercise training-induced regulation of mitochondrial quality. *Exercise and Sport Sciences Reviews*.

[29] Remels A. H. V., Langen R. C. J., Schrauwen P., Schaart G., Schols A. M. W. J., Gosker H. R. (2010). Regulation of mitochondrial biogenesis during myogenesis. *Molecular and Cellular Endocrinology*.

[30] Sin J., Andres A. M., Taylor D. J. R. (2015). Mitophagy is required for mitochondrial biogenesis and myogenic differentiation of C2C12 myoblasts. *Autophagy*.

[31] Zhu L. N., Ren Y., Chen J. Q., Wang Y. Z. (2013). Effects of myogenin on muscle fiber types and key metabolic enzymes in gene transfer mice and C2C12 myoblasts. *Gene*.

[32] Hughes S. M., Koishi K., Rudnicki M., Maggs A. M. (1997). MyoD protein is differentially accumulated in fast and slow skeletal muscle fibres and required for normal fibre type balance in rodents. *Mechanisms of Development*.

[33] Ryan M. T., Hoogenraad N. J. (2007). Mitochondrial-nuclear communications. *Annual Review of Biochemistry*.

[34] Scarpulla R. C. (2008). Transcriptional paradigms in mammalian mitochondrial biogenesis and function. *Physiological Reviews*.

[35] Baldelli S., Aquilano K., Ciriolo M. R. (2014). PGC-1*α* buffers ROS-mediated removal of mitochondria during myogenesis. *Cell Death & Disease*.

[36] Borrell R. M., Parker R., Henley E. J., Masley D., Repinecz M. (1980). Comparison of in vivo temperatures produced by hydrotherapy, paraffin wax treatment, and fluidotherapy. *Physical Therapy*.

